# Emotional distress and multimorbidity patterns in Chinese Han patients with osteoporosis: a network analysis

**DOI:** 10.3389/fpubh.2023.1242091

**Published:** 2024-01-11

**Authors:** Huiyao Wang, Qian Xia, Zaiquan Dong, Wanjun Guo, Wei Deng, Lan Zhang, Weihong Kuang, Tao Li

**Affiliations:** ^1^Mental Health Center, West China Hospital, Sichuan University, Chengdu, China; ^2^Department of Neurobiology, Affiliated Mental Health Center and Hangzhou Seventh People’s Hospital, Zhejiang University School of Medicine, Hangzhou, China; ^3^NHC and CAMS Key Laboratory of Medical Neurobiology, MOE Frontier Science Center for Brain Science and Brain-machine Integration, School of Brain Science and Brain Medicine, Zhejiang University, Hangzhou, China

**Keywords:** osteoporosis, multimorbidity, emotional distress, anxiety, depression

## Abstract

With the aging of the population, the prevalence of osteoporosis and multimorbidity is increasing. Patients with osteoporosis often experience varying levels of emotional distress, including anxiety and depression. However, few studies have explored the patterns of multiple conditions and their impact on patients’ emotional distress. Here, we conducted a network analysis to explore the patterns of multimorbidities and their impact on emotional distress in 13,359 Chinese Han patients with osteoporosis. The results showed that multimorbidity was prevalent in Chinese patients with osteoporosis and increased with age, and was more frequent in males than in females, with the most common pattern of multimorbidity being osteoporosis and essential (primary) hypertension. Finally, we found that patients’ emotional distress increased with the number of multimorbidities, especially in female patients, and identified eight multimorbidities with high correlation to patients’ emotional distress.

## Introduction

The prevalence of osteoporosis is increasing as the population of China ages. Osteoporosis is a systemic skeletal disease that is age-related and characterized by progressive loss of bone mass. It leads to a serious impairment in health-related quality of life and causes a high economic burden, especially in the older people, making it a major public health concern (1–3). Globally, the prevalence of osteoporosis is 23.1% in women and 11.7% in men among those aged 15–105 years (4). In China, the prevalence in osteoporosis is 20.6% in women and 5.0% in men among those over 40 years of age (5).

Patients with osteoporosis often experience varying levels of emotional distress, including anxiety and depression (6, 7). Studies have found that high levels of anxiety or depression may be associated with lower bone mineral density (6, 8, 9). This association may be due to excessive inflammatory responses that disrupt the normal metabolism of bone (10). Additionally, alterations in emotion-related hormone levels, such as cortisol, 25-hydroxyvitamin D and parathyroid hormone, may also contribute to bone loss and the development of osteoporosis (11, 12).

Moreover, the risk of multiple conditions, including hypertension, diabetes, coronary heart disease, and other chronic or acute diseases, also increases with age (13, 14), and multiple conditions may increase the risk of anxiety and depression (15–17). This makes the clinical treatment and management of osteoporosis more complex. A study in the United States found that women with osteoporosis have more comorbidities and depression than those without osteoporosis (18). However, among the world’s largest ethnic group, the Chinese Han, few studies have explored the patterns of multiple conditions and their impact on emotional well-being of patients with osteoporosis.

In this study, we utilized diagnostic and psychometric data obtained from a large general tertiary hospital in Southwest China to explore the patterns of multiple conditions and their impact on the emotional distress of Chinese Han patients with osteoporosis using network analysis. Our aim is to improve the understanding and management of osteoporosis in clinical settings.

## Methods

### Data source

For this study, we collected clinical data from the electronic medical records (EMR) of Chinese Han patients with osteoporosis aged 50 or older who were hospitalized in nonpsychiatric departments between January 1, 2015 and December 31, 2020. The dataset contained 25,999 records, of which 70.8% (18,417) were unique patients, and included patient demographics such as age, sex and marital status, and diagnostic information classified according to the 10th revision of the International Statistical Classification of Diseases (ICD-10). In this study, we extracted the first three characteristics of the ICD codes from EMR to classify the multiple conditions classification of osteoporosis. Ultimately, 669 disease codes were extracted, including a variety of codes for both chronic and acute diseases. According to VDA Harrison et al. (19), this concurrence of multiple chronic or acute conditions in a patient is defined as multimorbidity. Based on this definition, we named any disease other than osteoporosis as multimorbidity and counted the number of multimorbidities, with no multimorbidity counted as 0. Among these recodes, 13,359 (51.4%) patients underwent psychological evaluation using the Huaxi Emotional-distress Index (HEI) on a voluntary basis on admission for all non-psychiatric patients and were included in this analysis. The HEI is a self-reported nine-item scale widely used to screen emotional distress (anxiety and/or depression) in non-psychiatric clinical settings in China. It has demonstrated good reliability and validity and has been applied in online and offline survey and intervention studies (20–23). We defined a total score ≥ 11 as clinically significant anxiety and/or depression (CSAD) based on the optimal cutoff score for the HEI (20).

### Statistical analysis

Differences among groups were compared using *t*-test or χ^2^ test for quantitative or categorical variables, respectively. We evaluated risk factors for emotional distress in patients with osteoporosis, including sex, age, marital status, and number of multimorbidities from the EMR, by multivariable logistic regression analysis. We assessed statistical significance using a two-tailed alpha level of 0.05 with Stata v15.0 statistical software. To visualize variation, we created figures using EXCEL (Microsoft).

### Network analysis

In this study, we used a network analysis method to explore the multimorbidity patterns in patients with osteoporosis. This method is effective in analyzing and visualizing complex networks of diseases by identifying highly connected individual nodes and specific communities of nodes (13, 20, 23). Each node in the network represents a disease. Larger node diameters indicate higher prevalence, while the darker colors indicate more relationships with other diseases. The lines between nodes represent significant associations between diseases, while thicker lines indicating stronger associations. We used a relative risk to measure the correlations between disease pairs, calculated as the observed prevalence (O) divided by the expected prevalence (E) of the disease pair (24–26). For one disease pair composed of A and B, we computed the expected prevalence (E) as the prevalence of disease A multiplied by the prevalence of disease B, i.e., expected prevalence (E) = prevalence of disease A × prevalence of disease B. We considered a correlation significant if relative risk (O/E) was greater than 1.0. Using disease pairs with significant correlations, we constructed a network of multimorbidities and explore and visualize the network maps using Gephi 0.10, an open source software.

## Results

### Demographic and clinical characteristics in patients with osteoporosis

In this study, we included patients aged 50–101 years with an average age of 69.9 (SD = 10.2) years. Table 1 presents the demographic characteristics and emotional distress of the patients with osteoporosis. There were more female patients (10,717/13,359, 80.2%) than male patients (2,642/13,359, 19.8%). The prevalence of clinically significant anxiety and depression (CSAD) was higher among female patients, and married patients has the lowest CSAD rate. The prevalence of CSAD decreased with age but increased with the number of multimorbidities. All differences between groups were statistically significant (*p* < 0.05).

**Table 1:** Demographic characteristics and emotional distress of patients with osteoporosis.

Variables		*n* (%)	Emotional distress		*p* value
			Without CSAD^a^	With CSAD	
		(*n* = 13,359)	*n* (%)	*n* (%)	
			(*n* = 12,342)	(*n* = 1,017)	
**Sex**					
	Female	10,717 (80.2)	9,874 (92.1)	843 (7.9)	<0.001
	Male	2,642 (19.8)	2,468 (93.4)	174 (6.6)	
**Age (years)**					
	Mean (±SD)	69.9 (±10.2)	70.1 (±10.3)	68.4 (±9.8)	<0.001
**Marriage**					
	Married	11,598 (86.8)	10,750 (92.7)	848 (7.3)	0.006
	Unmarried	83 (0.6)	76 (91.6)	7 (8.4)	
	Widowed	1,474 (11.0)	1,335 (90.6)	139 (9.4)	
	Divorced	204 (1.5)	181 (88.7)	23 (11.3)	
**Number of multimorbidity**					
	Mean (±SD)	6.4 (±4.8)	6.3 (±4.7)	7.3 (±4.9)	<0.001

a CSAD, Clinically significant anxiety and/or depression.

### Distribution of multimorbidities

**Figure 1:**
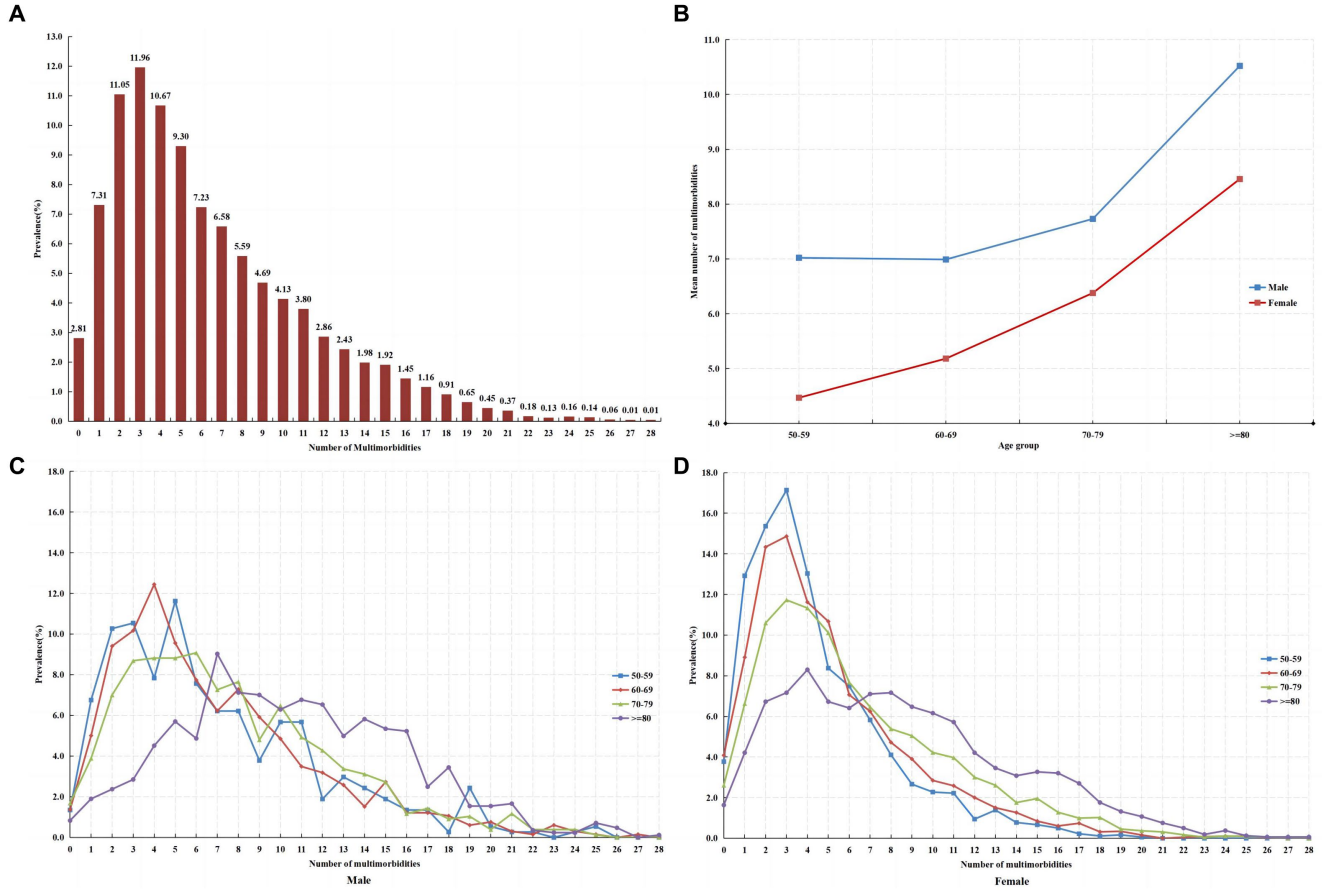
**(A)** The number of multimorbidities in patients with osteoporosis. **(B)** The average number of multimorbidities in patients with osteoporosis by age and sex. **(C)** The number of multimorbidities in male patients with osteoporosis by age group. **(D)** The number of multimorbidities in female patients with osteoporosis by age group.

As shown in Table 1 and Figure 1A, nearly all patients (97.2%) had at least one multimorbidity, with an average number of 6.4 (SD = 4.8) multimorbidities ranging from 0 to 28. The most common pattern was having three multimorbidities. The average number of multimorbidities was approximately 1.4 times higher in male patients (8.3 ± 5.3) than in female patients (5.9 ± 4.5), and the number of multimorbidities increased with age (Figure 1B). Among male patients, the highest number of multimorbidities was five at 50–59 years, four at 60–69 years, six at 70–79 years, and seven at ≥80 years (Figure 1C). In female patients, the highest number of multimorbidities was three in all age groups, except for those aged ≥80 years, who had four (Figure 1D). Overall, the number of multimorbidities in male patients declined slowly compared to that in female patients, suggesting that the pattern of multimorbidities in male patients with osteoporosis was more complex than that in female patients.

Table 2 summarizes the 16 separate multimorbidities with a prevalence over 10.0% in patients with osteoporosis, and two or more separate multimorbidities co-occurrence with each other are shown by network analysis. The most common separate multimorbidity was essential (primary) hypertension with code I10 (5,869, 43.9%) across all sex and age groups. Among osteoporosis, 14 separate multimorbidities showed a significantly different prevalence between female and male patients. In female patients, the second and third most common separate multimorbidities were other intervertebral disk disorders (M51) and gonarthrosis [arthrosis of knee] (M17), while non-insulin-dependent diabetes mellitus (E11) and other chronic obstructive pulmonary disease (J44) were the second and third most common separate multimorbidities in male patients. Furthermore, it is evident that for most multimorbidities, prevalence increases with age. In addition to essential (primary) hypertension (I10), in patients aged 50–69 years, diseases of liver (K76), other intervertebral disk disorders (M51), and gonarthrosis [arthrosis of knee] (M17) were the major multimorbidities. For those aged 70–79 years, non-insulin-dependent diabetes mellitus (E11), other intervertebral disk disorders (M51), and gonarthrosis [arthrosis of knee] (M17) were the predominant multimorbidities. Lastly, in patients aged 80 years and older, other chronic obstructive pulmonary disease (J44), atherosclerosis (I70), and heart failure (I50) emerged as the major multimorbidities.

**Table 2:** Comparison of separate multimorbidities with a prevalence over 10% in patients with osteoporosis by sex and age groups.

Code of disease^a^	Total	Sex group					Age group								
		Female		Male		*p* value	50–59		60–69		70–79		≥80		*p* value
	(*n* = 13,359)	(*n* = 10,717)		(*n* = 2,642)			(*n* = 2,173)		(*n* = 4,453)		(*n* = 4,300)		(*n* = 2,433)		
	*n* (%)	*n* (%)	Rank	*n* (%)	Rank		*n* (%)	Rank	*n* (%)	Rank	*n* (%)	Rank	*n* (%)	Rank	
I10	5,869 (43.9)	4,590 (42.8)	1	1,279 (48.4)	1	<0.001	504 (23.2)	1	1,591 (35.7)	1	2,220 (51.6)	1	1,554 (63.9)	1	<0.001
E11	2,691 (20.1)	1,964 (18.3)	4	727 (27.5)	2	<0.001	250 (11.5)	5	739 (16.6)	5	1,031 (24.0)	2	671 (27.6)	6	<0.001
M51	2,656 (19.9)	2,160 (20.2)	2	496 (18.8)	8	0.111	398 (18.3)	3	930 (20.9)	2	913 (21.2)	3	415 (17.1)	13	<0.001
I70	2,468 (18.5)	1,790 (16.7)	6	678 (25.7)	4	<0.001	221 (10.2)	8	618 (13.9)	6	883 (20.5)	5	746 (30.7)	3	<0.001
K76	2,462 (18.4)	1,904 (17.8)	5	558 (21.1)	6	<0.001	402 (18.5)	2	838 (18.8)	4	768 (17.9)	7	454 (18.7)	9	0.69
M17	2,331 (17.4)	2,098 (19.6)	3	233 (8.8)	16	<0.001	312 (14.4)	4	835 (18.8)	3	884 (20.6)	4	300 (12.3)	14	<0.001
N28	2,114 (15.8)	1,531 (14.3)	7	583 (22.1)	5	<0.001	236 (10.9)	7	545 (12.2)	7	806 (18.7)	6	527 (21.7)	8	<0.001
I67	1,890 (14.1)	1,430 (13.3)	8	460 (17.4)	10	<0.001	144 (6.6)	12	475 (10.7)	10	699 (16.3)	8	572 (23.5)	7	<0.001
I25	1,738 (13.0)	1,201 (11.2)	10	537 (20.3)	7	<0.001	72 (3.3)	15	276 (6.2)	15	678 (15.8)	9	712 (29.3)	5	<0.001
I50	1,651 (12.4)	1,163 (10.9)	13	488 (18.5)	9	<0.001	83 (3.8)	14	309 (6.9)	14	539 (12.5)	13	720 (29.6)	4	<0.001
J98	1,648 (12.3)	1,239 (11.6)	9	409 (15.5)	11	<0.001	197 (9.1)	10	474 (10.6)	11	536 (12.5)	14	441 (18.1)	12	<0.001
J44	1,606 (12.0)	916 (8.5)	16	690 (26.1)	3	<0.001	50 (2.3)	16	237 (5.3)	16	559 (13.0)	10	760 (31.2)	2	<0.001
M48	1,529 (11.4)	1,175 (11.0)	11	354 (13.4)	13	<0.001	203 (9.3)	9	502 (11.3)	9	552 (12.8)	11	272 (11.2)	15	<0.001
I51	1,479 (11.1)	1,129 (10.5)	14	350 (13.2)	14	<0.001	119 (5.5)	13	365 (8.2)	12	546 (12.7)	12	449 (18.5)	11	<0.001
E78	1,430 (10.7)	1,168 (10.9)	12	262 (9.9)	15	0.144	248 (11.4)	6	529 (11.9)	8	434 (10.1)	15	219 (9.0)	16	0.001
G47	1,386 (10.4)	1,020 (9.5)	15	366 (13.9)	12	<0.001	155 (7.1)	11	364 (8.2)	13	414 (9.6)	16	453 (18.6)	10	<0.001

a I10, Essential (primary) hypertension; E11, Non-insulin-dependent diabetes mellitus; M51, Other intervertebral disk disorders; I70, Atherosclerosis; K76, Other diseases of liver; M17, Gonarthrosis [arthrosis of knee]; N28, Other disorders of kidney and ureter, not elsewhere classified; I67, Other cerebrovascular diseases; I25, Chronic ischaemic heart disease; I50, Heart failure; J98, Other respiratory disorders; J44, Other chronic obstructive pulmonary disease; M48, Other spondylopathies; I51, Complications and ill-defined descriptions of heart disease; E78, Disorders of lipoprotein metabolism and other lipidaemias; G47, Sleep disorders.

### Risk factors for emotional distress in patients with osteoporosis

According to Table 1, there were differences in both demographic information (including sex, age, and marital status) and the number of multimorbidities for emotional distress, so the multivariable logistic regression analysis included all of these variables in the model for analysis. And, given that females and males would typically have different multimorbidity patterns, we conducted further analyses for the female and male groups separately. Table 3 presents the odds ratios of selected risk factors associated with emotional distress (CSAD) in patients with osteoporosis. The findings indicate that the female patients have a 1.23 times higher risk of experiencing CSAD compared to male patients. In female patients, widowhood has been identified as a significant risk factor for CSAD, and the risk of CSAD increases in correlation with the number of multimorbidities. These risk factors have not been identified in male patients. In addition, for both female and male patients, the risk of CSAD decreases with age.

**Table 3:** Risk factors for emotional distress (CSAD^a^) in patients with osteoporosis.

Variables		All patients (*n* = 13,359)			Female patients (*n* = 10,717)			Male patients (*n* = 2,642)		
		OR^b^	95% CI^c^	*p* value	OR	95%CI	*p* value	OR	95% CI	*p* value
**Sex**										
	Male	1			–	–	–	–	–	–
	Female	1.23	1.030–1.462	0.022	–	–	–	–	–	–
Age (years)		0.97	0.967–0.0980	<0.001	0.97	0.963–0.978	<0.001	0.98	0.970–0.997	0.014
**Marriage**										
	Married	1			1			1		
	Unmarried	1.07	0.488–2.327	0.873	0.88	0.353–2.211	0.790	2.11	0.466–9.542	0.333
	Widowed	1.52	1.244–1.853	<0.001	1.62	1.318–1.998	<0.001	0.68	0.294–1.593	0.379
	Divorced	1.41	0.905–2.195	0.129	1.37	0.850–2.199	0.197	1.76	0.515–6.009	0.368
Number of multimorbidity		1.06	1.045–1.073	<0.001	1.07	1.053–1.085	<0.001	1.02	0.993–1.054	0.131

a CSAD, Clinically significant anxiety and/or depression.

b OR, Odds ratio.

c CI, Confidence interval.

### Multimorbidity network of osteoporosis

**Figure 2:**
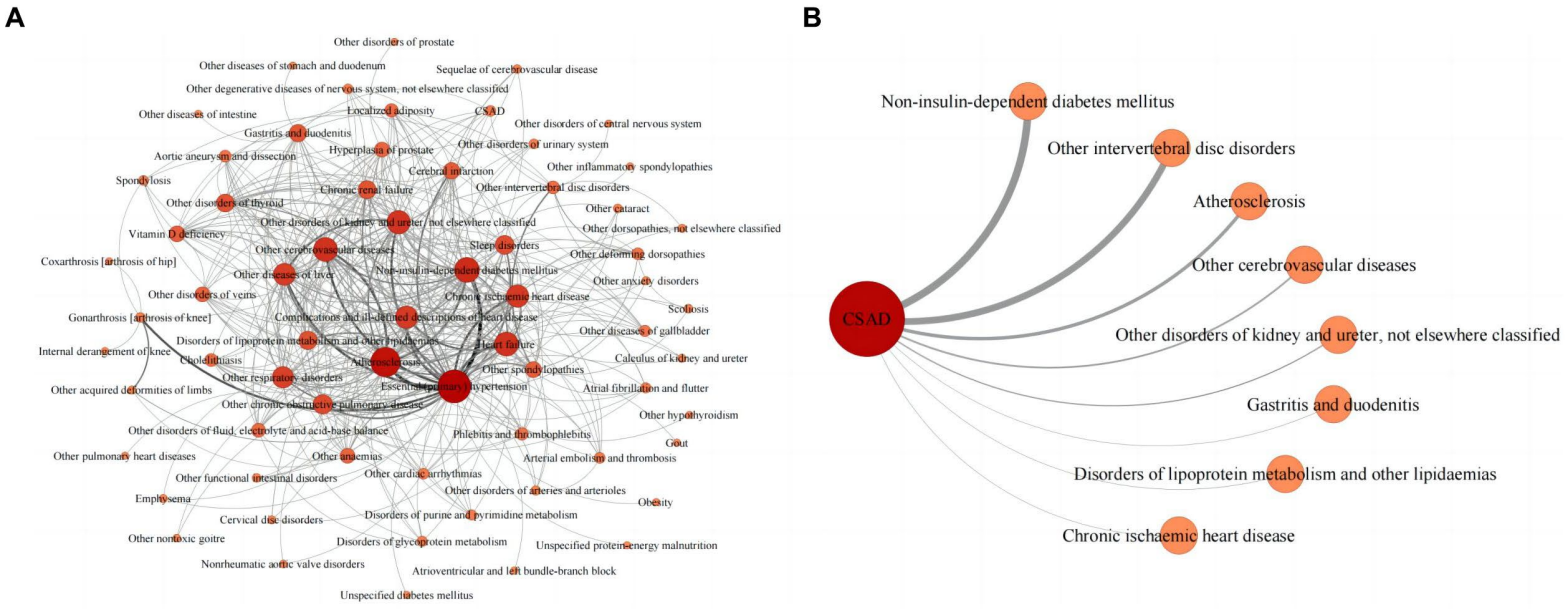
**(A)** Multimorbidity network in patients with osteoporosis. **(B)** CSAD* multimorbidity network in patients with osteoporosis. *CSAD, clinically significant anxiety and/or depression.

In this study, a total of 669 diseases were diagnosed alongside osteoporosis. Of these, diseases with a prevalence of multimorbidity greater than 1.0% and a relative risk (O/E) greater than 1.0 were incorporated into Gephi to create the osteoporosis multimorbidity map. This map consisted of 67 nodes, each representing a multimorbidity, and 407 links depicting the correlations between them, ultimately forming the osteoporosis multimorbidity network (Figure 2A).

The strongest link within this network connected essential (primary) hypertension (I10) and non-insulin-dependent diabetes mellitus (E11), with 1,835 co-occurrences. The next strongest link joined essential (primary) hypertension (I10) and atherosclerosis (I70), with 1,483 co-occurrences. The network’s three most prevalence categories were diseases of the circulatory system (e.g., essential (primary) hypertension, atherosclerosis, heart failure, other cerebrovascular diseases, and chronic ischemic heart disease), diseases of the musculoskeletal system and connective tissue (e.g., other spondylopathies, other intervertebral disk disorders, other deforming dorsopathies, gonarthrosis [arthrosis of knee], and other acquired deformities of limbs), and endocrine, nutritional and metabolic diseases (e.g., non-insulin-dependent diabetes mellitus, disorders of lipoprotein metabolism and other lipidemias, other disorders of thyroid, vitamin D deficiency, and localized adiposity). Essential (primary) hypertension (I10) and atherosclerosis (I70) served as the central hubs of the multimorbidity network. Additionally, the study identified eight multimorbidities that demonstrated a strong correlation with patients’ emotional distress. The most strongly correlated multimorbidity was non-insulin-dependent diabetes mellitus (E11), with 247 co-occurrences (Figure 2B).

## Discussion

In this study, we uncovered a high incidence of multimorbidities among patients with osteoporosis and identified a strong link between these multimorbidities and patients’ emotional distress. Utilizing network analysis, we determined common multimorbidities in osteoporosis and examined the interconnected relationships between them. Targeting these associated multimorbidities for specialized clinical interventions or clinical screening could benefit patients with osteoporosis.

The current study discovered that osteoporosis was more prevalent among female inpatients compared to male inpatients with osteoporosis. This sex disparity might be related to the higher occurrence of osteoporosis in females than in males. Previous studies, including those conducted in China, have also observed a higher prevalence of osteoporosis in females compared to males, which supports this observation (4, 5, 27).

In the evaluation of patients’ emotional distress, the prevalence of CSAD was found to be higher in female patients compared to male patients. Additionally, divorced, widowed, and unmarried patients exhibited a higher prevalence of CSAD than married patients. Regression analysis results indicated that female and widowhood were risk factors for CSAD, which aligns with previous findings that identified female, experiencing loss events, and poor social support as risk factors for anxiety or depression (28, 29).

Interesting, this study revealed that the prevalence of CSAD decreased with increasing age, and regression analysis identified advanced age as a protective factor for CSAD. Prior research on older adults suggests that this phenomenon may be due to improved emotional regulation and experience with age, as well as reduced stress reactivity to younger individuals, resulting in a smaller increase in negative emotions when faced with stressful events (30, 31). Similar phenomena have also been found in some disease-specific studies. For example, the study of irritable bowel syndrome (IBS) patients found that younger people had higher levels of anxiety and depression than older people (32), and the study of systemic lupus erythematosus (SLE) patients found that those who had the disease at a younger age had a higher risk of future depression (33). On the other hand, there were also some studies found that the risk of people suffering from anxiety or depression increases with age, as they experience more somatic illnesses and have less social support from important family members and friends (34–36). To our knowledge, few studies have examined this age-related difference in negative emotions among patients with osteoporosis. Furthermore, this study found that the patients with CSAD had a shorter average age and a higher average number of multimorbidities compared to patients without CSAD. These findings contribute additional evidence to the understanding of how emotional distress negatively impacts life expectancy and health status, highlighting the importance of addressing these issues during clinical treatment (37–40).

In analyzing multimorbidity among patients, this study observed that the number of multimorbidities increased with age, aligning with previous studies (41, 42). We also discovered that 97.2% of patients with osteoporosis had at least one multimorbidity, slightly higher than the results from a survey of osteoporosis patients in Germany, where over 95% of adults with osteoporosis had at least one comorbidity (1). This highlights high prevalence of multimorbidity among patients with osteoporosis, which requires attention. Moreover, we found that although the prevalence of osteoporosis was higher in female patients, male patients had more multimorbidities than that in female patients. This is consistent with the finding reported by Wang et al. (43), which reported a higher average number of multimorbidities in male inpatients in Chengdu, China. However, some studies have reported the opposite (41), suggesting that the inconsistency may be due to variations in observed diseases and the sample age ranges, which warrant further investigation.

In addition, this study found that female osteoporosis patients had a higher prevalence of CSAD and a lower number of multimorbidities compared to male osteoporosis patients. This is consistent with previous studies, which have found that females are more likely to suffer from anxiety or depression than males (44–46). Moreover, the results of the regression analysis showed that the number of multimorbidities significantly increased the risk of CSAD in female osteoporosis patients but not in male osteoporosis patients. This suggests that better management of multimorbidities in female patients would likely benefit female patients more.

Network analysis results demonstrated connections between osteoporosis and hypertension, diabetes, atherosclerosis, and other diseases across several systems, consistent with previous multimorbidity studies on osteoporosis (1, 41, 47). This supports the notion that osteoporosis is a systematic disease that often coexists with various common disorders in older individuals. Notably, this study was the first to identify eight somatic diseases strongly associated with emotional distress in patients with osteoporosis (Figure 2B). In clinical practice, focusing on the screening and management of these multimorbidities and implementing comprehensive intervention strategies can promote disease recovery and reduce emotional distress in patients with osteoporosis.

## Limitations

This study had several limitations. Firstly, as a single-center study, it relied on data from a large general tertiary hospital, with patients primarily from Southwest China, making the results regional-specific. Secondly, all data of the study were obtained from the EMR, which covers a wide range of disease types. Therefore, we assessed patients’ multimorbidity status using only disease counts, rather than using tools that include a limited number of disease categories that do not cover the types of diseases involved in this study, such as the Charlson Comorbidity Index, which includes 19 conditions and is widely used to measure patients’ comorbidity/multimorbidity status (48). Thirdly, due to the retrospective cross-sectional nature of the study based on patients’ hospitalization data, the sequence and risk factors for the occurrence of individual diseases were not investigated, and there was a deficiency of information on patients’ quality of life and disease prognosis, which made it unachievable to infer the causal relationship between individual diseases and different clusters of multimorbidity and to assess the specific impact of multimorbidity on patients. In light of these limitations, future research should investigate the quality of life and disease prognosis in patients with osteoporosis to better understand the impact of multimorbidity. Additionally, efforts should be made to analyze the differences in multimorbidities and to incorporate multimorbidity data to predict prognostic outcomes.

## Conclusion

This study examined emotional distress and investigated multimorbidities patterns using network analysis in patients with osteoporosis. The findings revealed that multimorbidities were prevalent among patients and increased with age. Significant differences in emotional distress and the majority of the multimorbidities were observed across sex and age groups. Emotional distress in patients decreased with age and increased with the number of multimorbidities, especially in female patients. The most common patterns of multimorbidity among patients with osteoporosis was osteoporosis and essential (primary) hypertension, while non-insulin-dependent diabetes, other intervertebral disk diseases and atherosclerosis were also major multimorbidities and strongly correlated with patients’ emotional distress. These results offer valuable insight for enhancing the prevention and management of multimorbidities and emotional distress in osteoporosis patients, ultimately contributing to an improved quality of life for those affected.

## Data availability statement

The raw data supporting the conclusions of this article will be made available by the authors, without undue reservation.

## Ethics statement

The studies involving humans were approved by the Ethics Committee of West China Hospital, Sichuan University, Chengdu, Sichuan, China. The studies were conducted in accordance with the local legislation and institutional requirements. The ethics committee/institutional review board waived the requirement of written informed consent for participation from the participants or the participants’ legal guardians/next of kin because The Ethics Committee waived the requirement for consent because the study is a retrospective analysis of routinely collected administrative data.

## Author contributions

HW, QX, and TL: conceptualization, methodology, validation, and writing—original draft. ZD, WG, WD, LZ, and WK: conceptualization, methodology, and writing—review. All authors contributed to the article and approved the submitted version.
